# Editorial: Inflammation in Cardiovascular Diseases: Role of the Endothelium and Emerging Therapeutics

**DOI:** 10.3389/fphar.2020.614387

**Published:** 2020-11-17

**Authors:** Cheng Xue Qin, Owen L. Woodman, Chen Huei Leo

**Affiliations:** ^1^ Drug, Discovery Biology, Monash Institute of Pharmaceutical Sciences, Monash University, Parkville, VIC, Australia; ^2^ Baker Heart & Diabetes Institute, Melbourne, VIC, Australia; ^3^ Science, Math and Technology, Singapore University of Technology & Design, Singapore, Singapore

**Keywords:** endothelial dysfunction, inflammation, cardiovascular disease, pharmacotherapeutics, vascular homeostasis and remodeling

Endothelial cells, lining the interior surface of all blood vessels, not only participate in the maintenance of the delivery of blood to all vital organs but are also involved in the maintenance of vascular homeostasis. Specifically, endothelial cells play an important role in physiological processes such as the control of vasomotor tone, angiogenesis, leukocyte trafficking, and both innate and adaptive immunity. A great bulk of evidence suggests that multiple diseases, such as atherosclerosis, ischemia, hypertension or diabetes have detrimental effect on endothelium, contributing to the development of cardiovascular diseases (CVD). One of the key common central mechanisms that links all of these diseases is an exaggerated inflammatory response within the endothelium. In all cases, the interaction between inflammatory cells and the endothelium plays a role crucial to the initiation of the pathological condition. Indeed, endothelial dysfunction often encompasses a pro-inflammatory endothelium, contributing to reduced vasodilation, and increased vascular stiffness. Therefore, the main goal of this Research Topic is to provide new mechanistic insights on (patho)physiological events driving inflammation within the endothelium in conditions of multiple diseases, including atherosclerosis, ischemia, hypertension and diabetes.

As a critical contributor to vascular health endothelial function is an important contributor to the regulation of vascular tone, platelet aggregation and leukocyte adhesion. It is well established that endothelial dysfunction is linked with cardiovascular disease and assessment of endothelial function, for example through the assessment of flow mediated vasodilatation, has become an important clinical tool in the prediction of adverse cardiovascular events (Xu et al., 2014). There are multiple endothelium-derived vasodilators such as nitric oxide (NO•), nitroxyl (HNO), hydrogen sulfide (H_2_S) and mediators of endothelium-dependent hyperpolarisation (EDH). Much attention has been paid to the impaired activity of NO•, due to the impaired activity of the synthetic enzyme endothelial nitric oxide synthase (eNOS) and its increased inactivation by oxidative stress, in cardiovascular disease (Förstermann and Münzel, 2006). Here Sun et al. and Velagic et al. review the less well documented roles of endothelium-derived H_2_S and HNO, respectively. It is suggested by Sun et al. that H_2_S may be an important inhibitor of endothelial inflammation suggesting a potential role for improved H_2_S donors as therapeutics for CVD. Unlike NO• and H_2_S, Velagic et al. discuss the preservation of responses to both endogenous and exogenous HNO in the presence of diabetes-induced oxidative stress and postulate that HNO donors may offer better efficacy than traditional NO-donors in the treatment of vascular disease. Tran et al. focus on metabolic syndrome as a precursor to diabetes and provide a thorough review of animal models that might be best employed to better understand the mechanisms of endothelial dysfunction, making the important point that the role of vasoconstrictor prostanoids is worthy of further investigation. The role of chemokine receptor 5 (CCR5) in metabolic disease and endothelial inflammation is explored by Zhang et al. who consider both the possibilities and difficulties of targeting CCR5 and its ligands in cardiovascular disease. The role of endothelial dysfunction in the genesis of age-related macular degeneration is considered by Yeo et al. who review the mechanisms of choroidal neovascularization and animal models that may advance knowledge in this area. In further consideration of vascular remodeling, Liu et al. investigate changes in pulmonary veins in a hypoxia pulmonary artery hypertension model in mice. In an elegant series of experiments it is demonstrated that hypoxia leads to inhibition of SERCA2 activity to promote calcium influx through the TRPC6 channel resulting in cell proliferation, migration and inhibition of apoptosis. Ding et al. review the rapidly developing area of endothelial and smooth muscle senescence as a contributor to vascular pathology and consider how epigenetic mechanisms may contribute to vascular inflammation and aging.

In the last 2–3 decades, many scientists have interrogated the mechanisms of action of the inflammatory signaling processes that are actively engaged to promote inflammation in the cardiovascular system. A great bulk of evidence suggests that multiple diseases, such as atherosclerosis, ischemia and diabetes, have detrimental effects on the endothelium, contributing to the development of CVD. One of the key common central mechanisms that links all of these diseases is an exaggerated inflammatory cytokine production and response.

The review by Ye et al. summarizes the role of interleukin-12 family members, a class of inflammatory cytokine, in their regulation and progression of various cardiovascular diseases, including atherosclerosis, hypertension, aortic dissection, cardiac hypertrophy, myocardial infarction, and acute cardiac injury. The authors highlight key knowledge gaps in the molecular and cellular mechanisms of interleukin-12 biology, and suggest that a better understanding of these disease processes is critical for the identification of possible targets for prevention which could lead to clinical treatment of a variety of cardiovascular diseases.

Sepsis-induced cardiomyopathy is one of the major predictors of morbidity and mortality of sepsis, present in more than 40% of cases of sepsis and its appearance can increase the mortality rate up to 70%. The review by Lin et al. provides a comprehensive summary of the recent progress in the pathophysiological characterization, diagnosis and current treatments (both pharmacological and non-drug) of septic cardiomyopathy. Furthermore, the authors also introduced several potential novel treatments for septic cardiomyopathy, which includes gene therapy, mitochondrial targeted therapy and inhibition of inflammatory mediators. In addition, the original study reported by Zhang et al. describes the protective actions of the novel anti-inflammatory agent, resolvin E1, an omega-3 polyunsaturated fatty acid-derived metabolite in an animal model of sepsis-induced cardiomyopathy. This exciting study reveals experimental evidence that resolvin E1 treatment inhibits mitogen-activated protein kinase (MAPK) and Nuclear factor kappa B (NF-κB) inflammatory signaling pathways, modulates macrophage polarization and reduces myocardial apoptosis leading to resolution of cardiac inflammation in sepsis-induced cardiomyopathy. This suggests that resolvin E1 may be a novel lipid mediator for the treatment of sepsis-induced cardiomyopathy.

Inflammation is also widely regarded as a key culprit for the pathogenesis of abdominal aortic aneurysm and diabetic cardiomyopathy. The original study by Yan et al. provides experimental evidence for bazedoxifene, a clinically approved therapy for the prevention and treatment of postmenopausal osteoporosis, as a potentially novel treatment for abdominal aortic aneurysm. In their study, Yan et al. indicated that bazedoxifene downregulated the IL-6/GP130/STAT3-dependent inflammatory signaling pathway and attenuated the formation of abdominal aortic aneurysm in angiotensin II-infused ApoE^−/−^ mice. In another original study by Zou et al., the team investigated the potential of a natural compound derived from a Chinese herb, sophocarpine to protect against diabetic cardiomyopathy. Specifically, *in vitro* and *in vivo* experiments revealed that sophocarpine treatment protected myocardial cells from hyperglycemia-induced injury by improving mitochondrial function, suppressing NF-κB-dependent inflammatory signaling pathways and inhibiting cardiac apoptosis. In addition to sophocarpine, other natural products such as flavonoids, are also widely studied as potential treatment for cardiovascular diseases in the context of various disease settings including diabetes. In this regard, Choy et al. elegantly reviewed the therapeutic potential of flavonoids by acting as natural anti-inflammatory agents which target NF-κB inflammatory signaling pathways. The review provides a comprehensive update of the mechanisms underlying NF-κB-induced inflammation in various cardiovascular pathologies and discusses how flavonoids may inhibit the activation of NF-κB and mitigate the inflammatory responses in these disease processes.

Myocardial ischemia/reperfusion injury (I/R) is a complex and multifactorial pathophysiological process in which excess oxidative stress and inflammatory response are essential to the development of both acute and long-term consequences after the ischemic insult. This initial insult to the myocardium is often followed by a pro-inflammatory phase, a proliferative phase and a subsequent remodeling phase. Thus, the development of effective pharmacotherapies, especially by targeting the oxidative stress, inflammatory and remodeling pathways, may improve the clinical outcome of patients in cardiac emergency (Heusch, 2020).

Zhang et al. demonstrated that activation of aldehyde dehydrogenase 2 (ALDH2) is cardioprotective against post-cardiac arrest myocardial dysfunction by attenuating mitochondrial ROS in a rat cardiac arrest model. These observations provided novel evidence for the role of excess aldehyde-induced ROS production in the mitochondria, suggesting that therapeutic targeting of ALDH2 may be an innovative approach for treating post-cardiac arrest-induced myocardial dysfunction. Yin et al. investigated whether overexpression of inhibitor of differentiation 2 (Id2, a transcriptional repressor) could preserve cardiac function and ameliorate cardiac fibrosis and apoptosis through modulation of TGF-β1/Smad3/hypoxia induced factor-1 alpha (HIF-1α)/interleukin (IL)-11 pathway. These observations suggest that Id2 could provide another novel target for cardiac fibrosis after myocardial infarction. Liu et al. evaluated the efficacy and safety of AFC1, a novel derivative from tanshinone IIa (a natural compound derived from Salvia miltiorrhiza) in a mouse model of myocardial ischemia/reperfusion (I/R) injury, likely by reducing platelet-derived growth factor receptors (PDGFR) and STAT signaling. This result confirmed that AFC1 exerts anti-hypertensive and anti-fibrotic effects against myocardial I/R injury and suggest that AFC1 may be a novel approach for patients suffering myocardial I/R injury.

Finally, we wish to highlight that this wealth of knowledge regarding the breadth of the impact of vascular inflammation in this Research Topic, which has provided new mechanistic insights on (patho)physiological events driving inflammation within the endothelium in CVD, including atherosclerosis, ischemia, hypertension, sepsis or diabetes. Emerging therapeutic strategies (small molecules, peptides, medical devices, natural products) that specifically target the inflammatory pathways or processes in the endothelium have been highlighted. In conclusion, deep knowledge on the endothelium in the cardiovascular system could accelerate the development of novel pharmacotherapies for cardiovascular disease.

## Author Contributions

CQ and CL conceived and designed the special issue. CXQ, OW, and CL drafted, edited and revised the manuscript. All authors approved the final version of the manuscript.

## Funding

CXQ received a National Heart Foundation Future Leader Fellowship.

## Conflict of Interest

The authors declare that the research was conducted in the absence of any commercial or financial relationships that could be construed as a potential conflict of interest.

## References

[B1] Förstermann U. Münzel T. (2006). Endothelial nitric oxide synthase in vascular disease: from marvel to menace. Circulation 13, 1708–1714. 10.1161/CIRCULATIONAHA.105.602532 16585403

[B2] Heusch G. (2020). Myocardial ischaemia–reperfusion injury and cardioprotection in perspective. Nat. Rev. Cardiol. 10.1038/s41569-020-0403-y 32620851

[B3] Xu Y. Arora R. C. Hiebert B. M. Lerner B. Szwajcer A. McDonald K. (2014). Non-invasive endothelial function testing and the risk of adverse outcomes: a systematic review and meta-analysis. Eur. Heart J. Cardiovasc. Imaging 15, 736–746. 10.1093/ehjci/jet256 24399339

